# Untargeted metabolomics reveals alternations in metabolism of bovine mammary epithelial cells upon IFN-γ treatment

**DOI:** 10.1186/s12917-023-03588-2

**Published:** 2023-02-11

**Authors:** Fengyang Li, Xiuhong Hu, Zengshuai Wu, Qiulei Yang, Qila Sa, Wenbo Ren, Tingting Wang, Zhengchao Ji, Na Li, Jing Huang, Liancheng Lei

**Affiliations:** 1 State Key Laboratory for Zoonotic Diseases, College of Veterinary Medicine, Jilin University, 1977 Xinzhu Road, Changchun, 130062 China; 2 Department of First Hospital, Jilin University, 1 Xinmin Street, Changchun, 130021 China; 3 Shannan Hospital, Shannan, 856099 China

**Keywords:** IFN-γ, Arginine depletion, Malignant transformation, Bovine mammary epithelial cells, Metabolomics

## Abstract

**Background:**

IFN-γ is a pleiotropic cytokine that has been shown to affect multiple cellular functions of bovine mammary epithelial cells (BMECs) including impaired milk fat synthesis and induction of malignant transformation via depletion of arginine, one of host conditionally essential amino acids. But the molecular mechanisms of these IFN-γ induced phenotypes are still unknown.

**Methods:**

BMECs were treated with IFN-γ for 6 h, 12 h, and 24 h. The metabolomic profiling in BMECs upon IFN-γ induction were assessed using untargeted ultra-performance liquid chromatography-mass spectrometry (UPLC-MS) metabolomic analysis. Key differentially expressed metabolites (DEMs) were quantified by targeted metabolomics.

**Results:**

IFN-γ induction resulted in significant differences in the contents of metabolites. Untargeted analysis identified 221 significantly DEMs, most of which are lipids and lipid-like molecules, organic acids and derivatives, organ heterocyclic compounds and benzenoids. According to Kyoto Encyclopedia of Genes and Genomes (KEGG) pathway analysis, DEMs were enriched in fructose and mannose metabolism, phosphotransferase system (PTS), β-alanine metabolism, arginine and proline metabolism, methane metabolism, phenylalanine metabolism, and glycolysis/gluconeogenesis. Quantification of selected key DEMs by targeted metabolomics showed significantly decreased levels of D-(-)-mannitol, argininosuccinate, and phenylacetylglycine (PAG), while increased levels in S-hydroxymethylglutathione (S-HMG) and 2,3-bisphospho-D-glyceric acid (2,3-BPG).

**Conclusions:**

These results provide insights into the metabolic alterations in BMECs upon IFN-γ induction and indicate potential theoretical basis for clarifying IFN-γ-induced diseases in mammary gland.

**Supplementary Information:**

The online version contains supplementary material available at 10.1186/s12917-023-03588-2.

## Introduction

Arginine is a conditional essential amino acid depending on the growth stage and body condition. For young mammals, arginine is one of the essential amino acids because of its low content in the breast milk and insufficient arginine synthesis [1]. On the other hand, endogenous synthesis of arginine can meet the demand of body's basal metabolism which makes it is a non-essential amino acid for adults. As the most abundant nitrogen carrier in proteins, arginine is the substrate for the synthesis of multiple substances including proteins, urea, ornithine, nitric oxide (NO), creatine, polyamines, nucleotides, proline, and agmatine [2, 3]. Thus, arginine plays vital roles in many biologic processes, including nutrients metabolism, cell growth and proliferation, release of hormone, and immune responses [4, 5].

In pathological conditions, cells under various stress factors are accompanied by abnormal arginine metabolism, resulting in significant reduction of intracellular arginine or arginine depletion. Many studies have found that arginine depletion is closely associated with the development of many diseases, such as cancer and infection [6, 7]. Arginine is required for tumor cell growth and actually, many tumor types including human breast cancer are arginine auxotrophic [8, 9], which makes arginine depletion an efficient therapeutic strategy for auxotrophic cancer treatment. However, a study by Cao et al. demonstrates that arginine supplementation inhibits the growth of breast cancer cells by enhancing innate and adaptive immune responses that are mediated by myeloid-derived suppressor cells in vivo [10]. Moreover, arginine also contributes to host immune defense in response to pathogenic infections. Arginine supplementation reduces the inflammatory response and susceptibility to *Staphylococcus aureus* (*S. aureus*) of bovine mammary epithelial cells (BMECs) and protects host from mastitis in vivo [11].

The influencing factors and mechanism of arginine depletion are poorly studied. Interestingly, arginine metabolism is influenced by IFN-γ, a pleiotropic inflammatory cytokine which usually involves in inflammation and autoimmune diseases [12, 13]. It’s found that the level of IFN-γ was closely associated with arginine depletion, suggesting IFN-γ possibly disorders arginine metabolism and leads to occurrence of diseases. Mice fed gluten-containing standard diet shows elevated IFN-γ level which may contribute to the higher type 1 diabetes incidence [14]. Arginine depletion reduces the expression of natural killer (NK) cell receptors and intracellular production of IFN-γ, which hinder NK cell functions [15]. Studies from our group demonstrate that IFN-γ induces arginine depletion that increase susceptibility to *S. aureus* and mastitis occurrence, impairs milk fat and protein synthesis, and malignant transformation of bovine mammary epithelial cells (BMECs) [11, 16, 17]. These results suggest that the regulation of arginine by IFN-γ might be therapeutic targets of some diseases. However, the exact mechanism for IFN-γ-induced arginine depletion of cells including BMECs is still unknown.

Metabolomics is a widely used tool to analyze the changes of various small molecule metabolites in systematic and molecular biology [18, 19]. Using high-throughput in silico analysis of metabolomics data, characteristic differentially expressed metabolites (DEMs) which reflect the functional status of living organisms can be identified without discrimination [20]. In this study, an untargeted and targeted metabolomics approach involving ultra-performance liquid chromatography-mass spectrometry (UPLC-MS) was applied to explore the mechanism underlying how IFN-γ induces arginine depletion of BMECs. All DEMs upon IFN-γ induction were identified and enriched by Kyoto Encyclopedia of Genes and Genomes (KEGG) pathway analysis [21–23]. Our study not only contributes to better understanding the arginine metabolism of BMECs, but also provides a molecular basis for the occurrence and prevention of diseases associated with IFN-γ-induced abnormal arginine metabolism.

## Materials and methods

### Chemicals and reagents

Bovine IFN-γ was purchased from the Kingfisher Biotech (S. Paul, MN, USA). LC–MS grade methanol and formic acid (98%) were bought from Sigma-Aldrich (St. Louis, MO, USA); acetonitrile was bought from Thermo (Shanghai, China). Ultrapure water was obtained with a Milli-Q system (Millipore Co., MA, USA). All chemicals and solvents used were of analytical or HPLC grade.

### Sample preparation for untargeted metabolic analysis

The BMECs cell line, MAC-T [24] (provided by Prof. Guoqiang Zhu, Yangzhou University, Yangzhou, China), was used in this study. All the cells were grown in Dulbecco’s modified Eagle’s medium/nutrient mixture F-12 (DMEM/F12) with 10% fetal bovine serum (FBS, CLARK, China), with 100 U/mL penicillin, 100 mg/mL streptomycin and incubated at 37 °C in a humidified atmosphere with 5% CO_2_. When entered the logarithmic growth phase, the cells were digested using 0.25% trypsin. For untargeted metabolomics detection, cells were treated with IFN-γ for different time periods 6 h, 12 h, and 24 h. After trypsin digestion and centrifugation at 1000 rpm for 10 min, the cell pellets were harvested and re-suspended in 1 mL pre-cooled PBS, followed by addition of 5 mL pre-cooled quenching reagent (8.6 g/L NH_4_HCO_3_ pH = 7.4, 60% ethanol), mixed evenly and centrifuged at 6000 g for 15 min at 4 °C. The cell pellets were re-suspended in 2 mL ultra-pure water and were sonicated in an ice bath for 10 min (5 s sonication with 5 s interval). The protein precipitation reagent (methanol: acetonitrile: water = 2: 2: 1, v/v/v) was added, mixed uniformly and centrifuged at 13,000 g, 4 °C for 15 min. The supernatant was collected for further analysis.

### Quality control (QC) sample preparation for untargeted metabolic analysis

Quality control samples (QC) were prepared by taking out same amount (50 μL) of volume from each group of samples. A total of 200 μL of each sample were dried and concentrated on a nitrogen blower at 37 °C, and dissolved in 50 μL of protein precipitation reagent in ampoules for further test.

### Sample detection by UPLC-MS for untargeted metabolic analysis

Liquid chromatography was performed with ExionL CAD UPLC equipped with a TripleTOF 5600 MS system. The samples were separated by an ACQUITY UPLC HSS T3 column (2.1 × 100 mm, 1.8 µm) at 35 °C, the injection volume was 5.0 μL. Mobile phase A: 0.1% formic acid; mobile phase B: 95% acetonitrile. Samples elution was performed at a flow rate of 0.35 mL/min. Gradient elution procedure was as follows: 0 - 0.5 min, 2% B; 0.5 -1.5 min, 2% - 20% B; 1.5 - 4.0 min, 20% - 65% B; 4.0 - 11.0 min, 65% - 95% B; 11.0 min - 15.0 min, 95% B; 15.0 min - 15.1 min, 95% - 2% B; 15.1 min - 20.0 min, 2% B. Samples were detected by electrospray ion source in both positive and negative ion modes. Samples were scanned with following parameters: DP = 100 V, CE = 35 eV, 100 - 1000 Da, curtain gas (CUR) = 30 psi, atomizing gas (GS1) = 55 psi, heating gas (GS2) = 55 psi, ion spray voltage (ISVF) = 5500 V, ion source temperature = 550 °C.

### Sample preparation for targeted metabolic analysis

Sample preparation for targeted metabolomics was performed as previously reported [25]. Reagents and materials for targeted metabolomics analysis are the same as those for nontargeted metabolomics. Briefly, the metabolite standards and the internal standards were dissolved with 90% acetonitrile to a final concentration of 5 mg/mL and 1 mg/mL, respectively. 50 μL samples were mixed with 50 μL internal standard working solution, 50 μL ethyl water (acetonitrile: water = 1:1, v/v), and 350 μL acetonitrile containing 1% formic acid. The mixture was vortex-mixed and centrifuged for 10 min at 4 °C, 12,000 rpm. The supernatant was collected for further analysis by UPLC-MS.

### Sample detection by UPLC-MS for targeted metabolic analysis

Liquid chromatography was performed with ExionL CAD UPLC equipped with a TripleTOF 5600 MS system. The samples were separated by a ZORBAX Eclipse XDB-C8 column (4.6 × 150 mm, 5 μm) at 30 °C, the injection volume was 15.0 μL. Mobile phase A: 0.1% formic acid; mobile phase B: 95% acetonitrile. Samples elution was performed at a flow rate of 0.2 mL/min. Gradient elution procedure is as follows: 0 - 2.5 min, 2% B; 2.5 - 4.0 min, 2% - 50% B; 4.0 - 6.0 min, 50% B; 6.0 - 6.1 min, 50% - 5% B; 6.1 min - 9.9 min, 5% B; 9.9 min - 10.0 min, 2% B. Samples were detected by electrospray ion source in positive ion mode. Samples were scanned with following parameters: DP = 60 V, CE = 10 eV, 50 - 250 Da, curtain gas (CUR) = 30 psi, atomizing gas (GS1) = 50 psi, heating gas (GS2) = 50 psi, ion spray voltage (ISVF) = 5500 V, ion source temperature = 500 °C.

### Data processing and analysis

The MS raw data were analyzed by Progenesis QI 2.3 software (Nonlinear Dynamics, WatersCorp, Durham, USA). Data of different samples were aligned according to the retention time deviation of 0.2 min and the mass deviation of 5 ppm, and analyzed according to the coefficient of variation (CV) value of 30%, signal-to-noise ratio of 3, minimum signal strength of 100,000. The significant differences of metabolites were verified by variable weight value (VIP) > 1.0, fold change (FC) > 2.0 or FC < 0.5 and *p* < 0.05. Experimental data were statistically analyzed by GraphPad (version 9.0). Data were presented as the mean ± standard deviation (SD) from three independent replicates. The differences between the mean values of normally distributed data were assessed by one-way ANOVA (Dunnett’s test). *p* < 0.05 was considered as statistically significant, which was indicated by "*". *p* < 0.01 indicated that the difference was extremely significant, which was indicated by "**".

## Results

### Detection of metabolites

To evaluate the metabolic alternations induced by IFN-γ in BMECs, we detected the metabolites in samples of IFN-γ treatment group and control group at different time (6 h, 12 h and 24 h) by untargeted metabolomics analysis in both negative and positive ion modes. Validation of the analytical method was achieved using quality control (QC) samples. The correlation heat map showed that the correlation coefficient among QC samples in both ionization modes was almost 1.0 (from 0.983 to 1.000) (Fig. S1), which indicates that the detection method has good stability and reproducibility of QC samples. According to the qualitative metabolite results, a total of 4,762 substance peaks (including 2,543 negative ion peaks and 2,219 positive ion peaks) in samples were detected. Furthermore, 2,253 annotated metabolites, including 976 negative ion mode metabolites and 1,277 positive ion mode metabolites, were identified.

### Multivariate data analysis

**Fig. 1:**
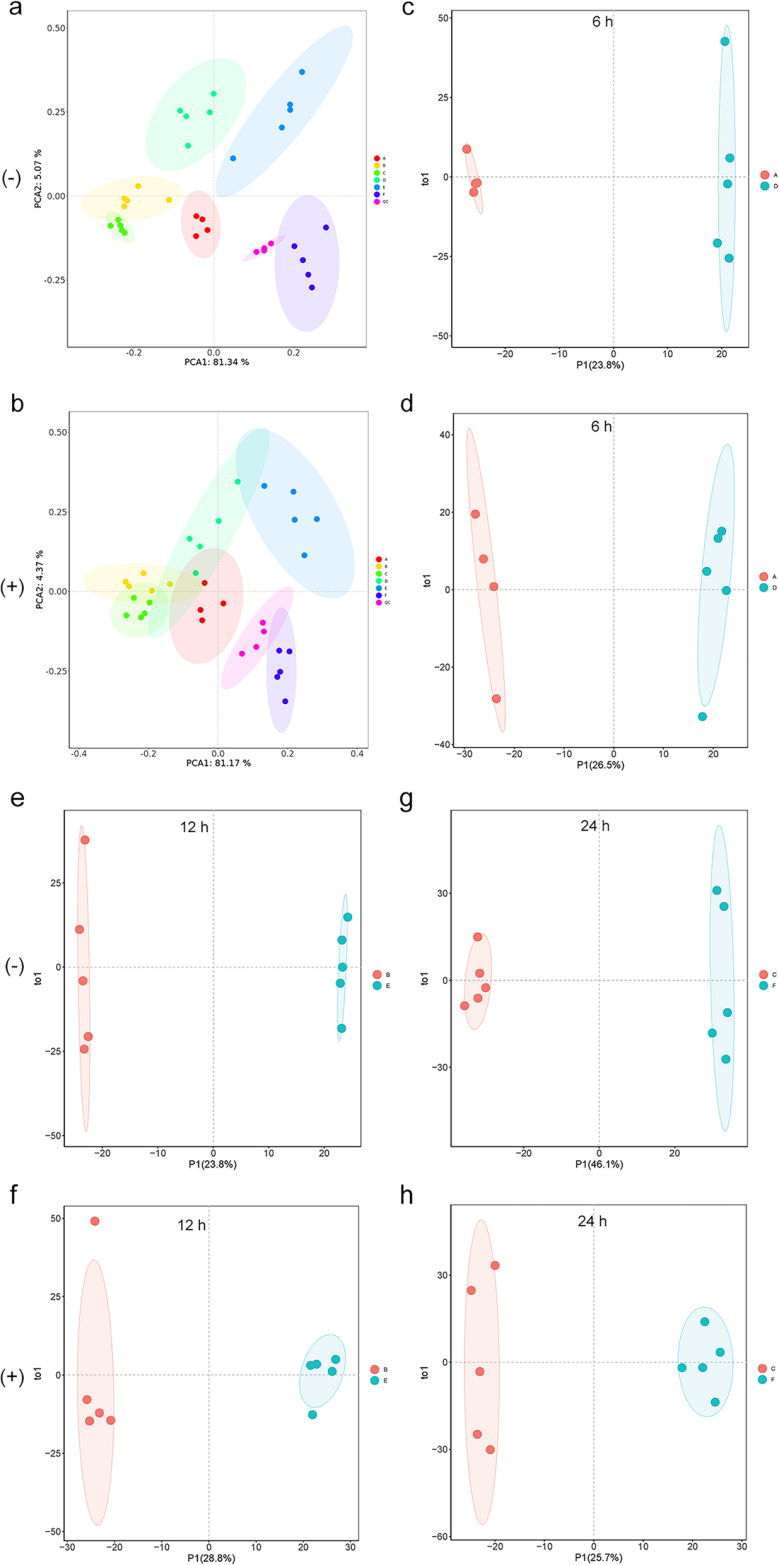
Multivariate statistical analysis of samples in positive and negative ion modes. Principal components analysis (PCA) in negative (**a**) and positive (**b**) ion modes; orthogonal partial least-squares discriminant analysis (OPLS-DA) score chart in negative (**c, e, g**) and positive (**d, f, g**) ion modes. Figure c, e, and g: control cells incubated for 6 h, 12 h, and 24 h, respectively; d, f, and h: cells stimulated with IFN-γ for 6 h, 12 h, and 24 h, respectively. (-), negative ion mode. ( +), positive ion mode. QC, quality control

Next, unsupervised principal component analysis (PCA) and orthogonal partial least squares discriminant analysis (OPLS-DA) was utilized to discriminate the overall distribution among samples. In the negative ion mode, the first and second principal components (PCA1 and PCA2) accounted for 81.34% and 5.07% of the total variance, respectively. While in the positive ion mode, the first and second principal components (PCA1 and PCA2) accounted for 81.17% and 4.37% of the total variance, respectively. The PCA showed clear differences among the groups in both ion modes, especially before and after IFN-γ induction (Fig. 1a and b). As time grows, the differences among control groups and IFN-γ induction groups, and the differences between control group and IFN-γ induction group gradually expanded, indicating there may have been significant differences in the expression of metabolites upon IFN-γ induction in BMECs. Similarly, the OPLS-DA model also displayed clear segmentation between the two groups in both ion modes (Fig. 1c-h). The permutation test of the OPLS-DA model showed that the interpretation rate (R^2^Y(CUM)) for the sample was close to 1 and the predictive ability (Q^2^(CUM)) was greater than 0.47 (Fig. S2), indicating that the model was reliable and can better explain and predict the differences in samples among the groups.

### Identification of differentially expressed metabolites

**Fig. 2:**
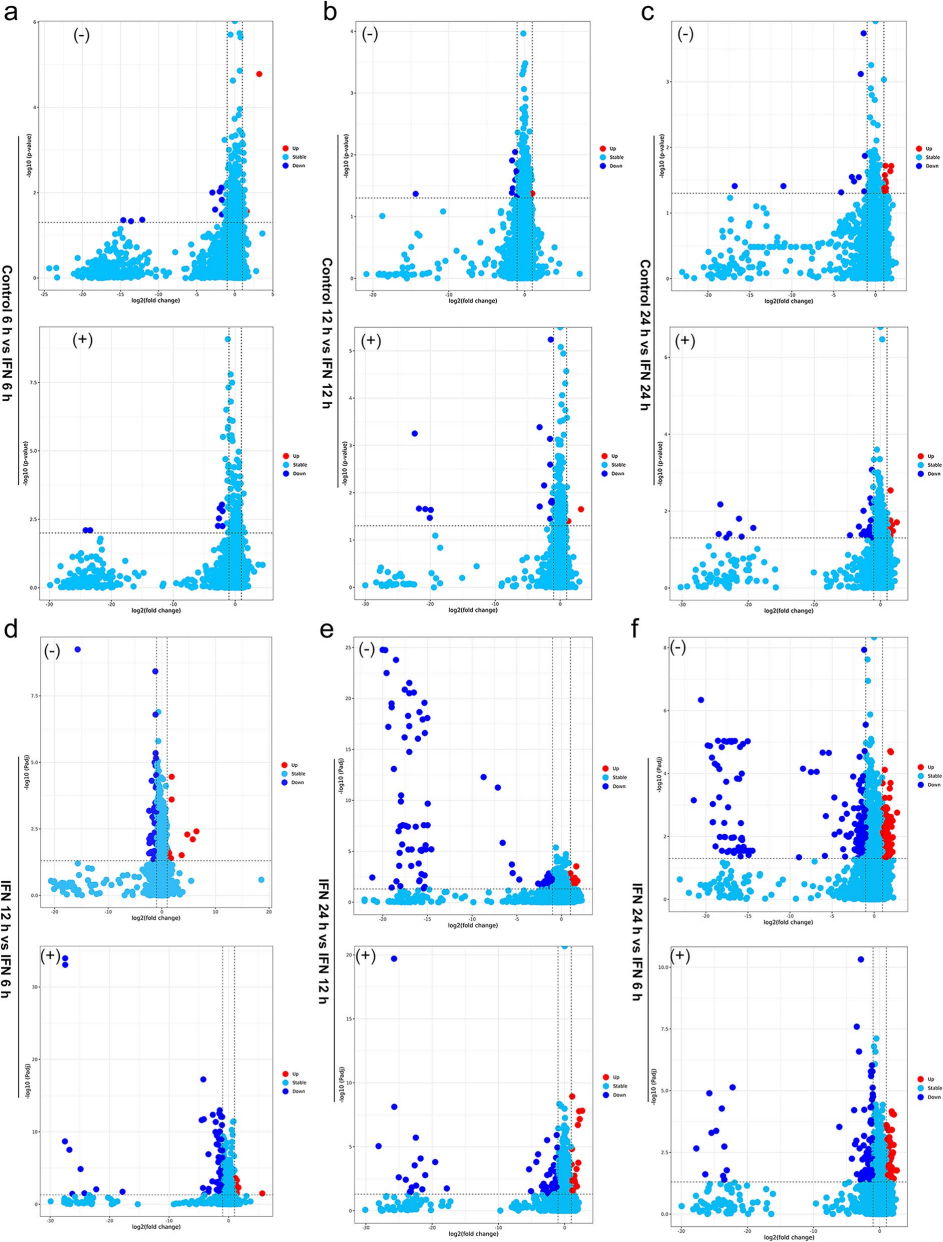
Volcano plots for the differentially expressed metabolites (DEMs) in negative and positive ion modes. (**a**) control (6 h) versus IFN-γ stimulation (6 h); (**b**) control (12 h) versus IFN-γ stimulation (12 h); (**c**) control (24 h) versus IFN-γ stimulation (24 h); (**d**) IFN-γ stimulation (12 h) versus IFN-γ stimulation (6 h); (**e**) IFN-γ stimulation (24 h) versus IFN-γ stimulation (12 h); (**f**) IFN-γ stimulation (24 h) versus IFN-γ stimulation (6 h). Downregulated or upregulated genes were divided by |log2Ratio|≥ 1 with false discovery rate (FDR) ≤ 0.01. Red dots for upregulated genes and dark blue dots for downregulated genes. vs, versus. (-), negative ion mode. ( +), positive ion mode

**Fig. 3:**
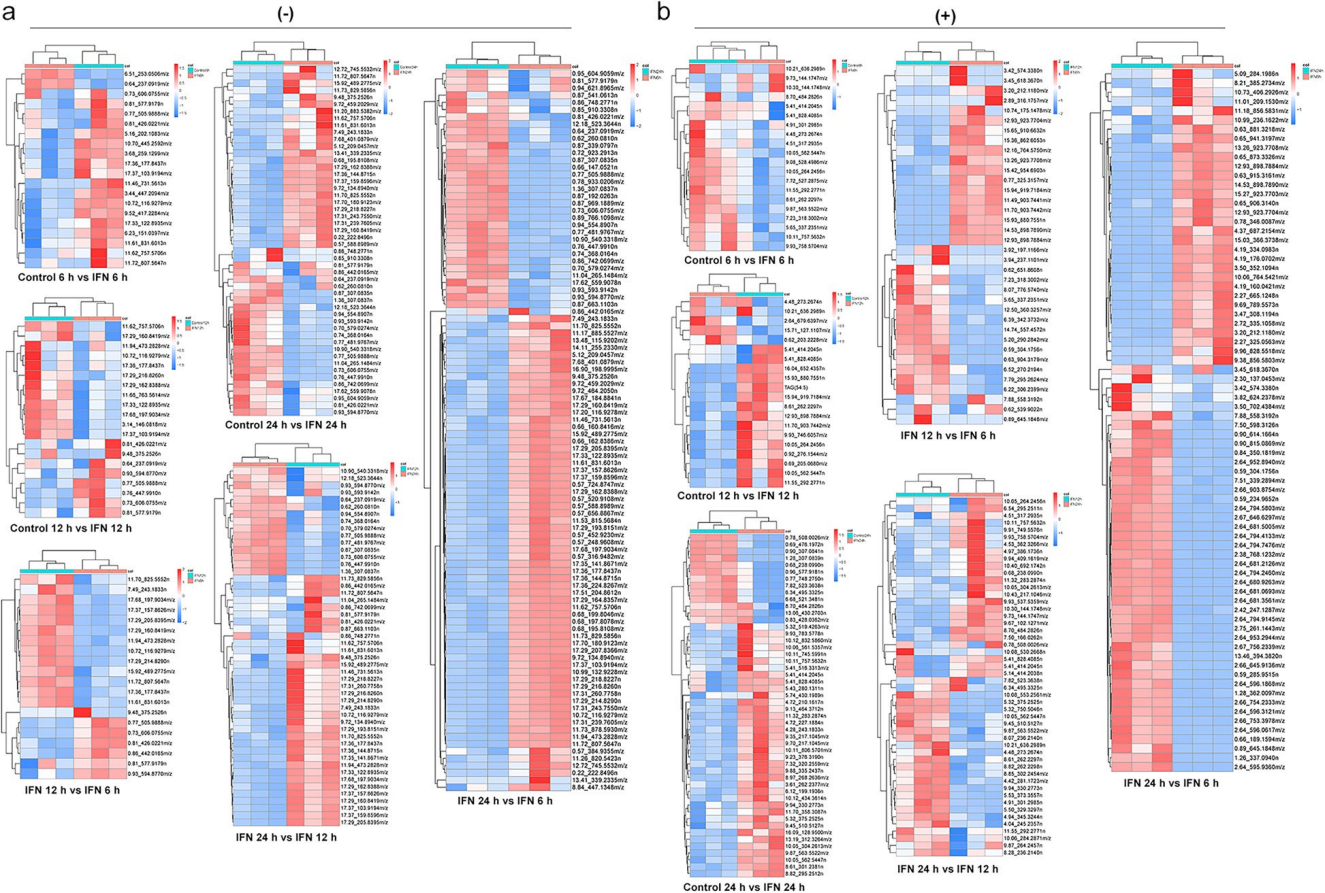
Heatmap of the differentially expressed metabolites (DEMs) in negative (**a**) and positive (**b**) ion modes. DEGs were screened out according to |log2Ratio|≥ 1 with false discovery rate (FDR) ≤ 0.01. Red rectangles indicate upregulated metabolites and blue rectangles indicate downregulated metabolites. (-), negative ion mode. ( +), positive ion mode

**Fig. 4:**
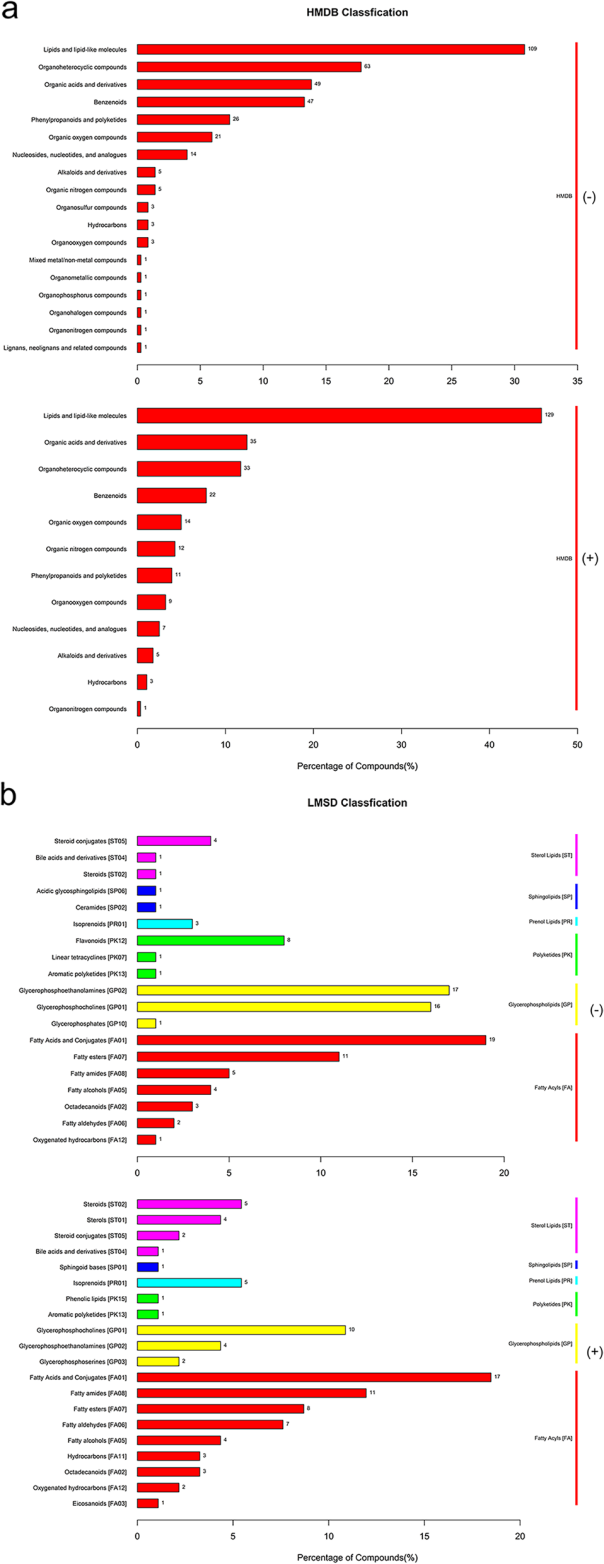
HMDB (**a**) and LMSD (**b**) classifications of the different metabolites of BMECs upon IFN-γ treatment. The Y-axis indicates the HMDB/LMSD terms. The number and degree of enrichment of genes in a category is displayed in the X-axis. The *p*-value < 0.05 is defined significant. (-), negative ion mode. ( +), positive ion mode. Color code: cyan, control group; red, IFN-γ treatment group

To further identify the differentially expressed metabolites (DEMs) in the samples, we screened the metabolites using variable importance projection (VIP) > 1, fold change (FC) > 2.0 or FC < 0.5 and *p-*value < 0.05 in the OPLS-DA model. After data acquisition and analyzation, a total of 108 and 113 DEMs were screened out in negative and positive ion modes, respectively (Table S1). Compared to control, there were 11 significantly DEMs (1 upregulated, 10 downregulated) upon IFN-γ treatment for 6 h (Table S2; Fig. 2a); 14 significantly DEMs (3 upregulated, 11 downregulated) upon IFN-γ treatment for 12 h (Table S3; Fig. 2b); and 25 significantly DEMs (14 upregulated, 11 downregulated) upon IFN-γ treatment for 24 h (Table S4; Fig. 2c). Compared to IFN-γ treatment for 6 h group, there were 27 significantly DEMs (11 upregulated, 16 downregulated) upon IFN-γ treatment for 12 h (Table S5; Fig. 2d); 86 significantly DEMs (37 upregulated, 49 downregulated) upon IFN-γ treatment for 24 h (Table S6; Fig. 2e). Compared to IFN-γ treatment for 12 h group, there were 58 significantly DEMs (13 upregulated, 45 downregulated) upon IFN-γ treatment for 24 h (Table S7; Fig. 2f). The volcano plots between each comparison group displayed the DEMs that contributed to the sample separation (Fig. 2a-f). Subsequently, we applied hierarchical clustering analysis and found that DEMs were distinguishable in the heat map (Fig. 3a and b). According to the Human Metabolome Database (HMDB), the majority of different metabolites were lipids and lipid-like molecules; organic acids and derivatives; organ heterocyclic compounds; benzenoids; phenylpropanoids and polyketides; and organic oxygen compounds (Fig. 4a). Furthermore, the majority of identified lipids belong to fatty acid conjugates, fatty amides, glycerophosphocholines, glycerophosphoethanolamines, and fatty esters according to the LIPID MAPS Structure Database (LMSD) classification (Fig. 4b).

### KEGG pathway enrichment analysis

**Fig. 5:**
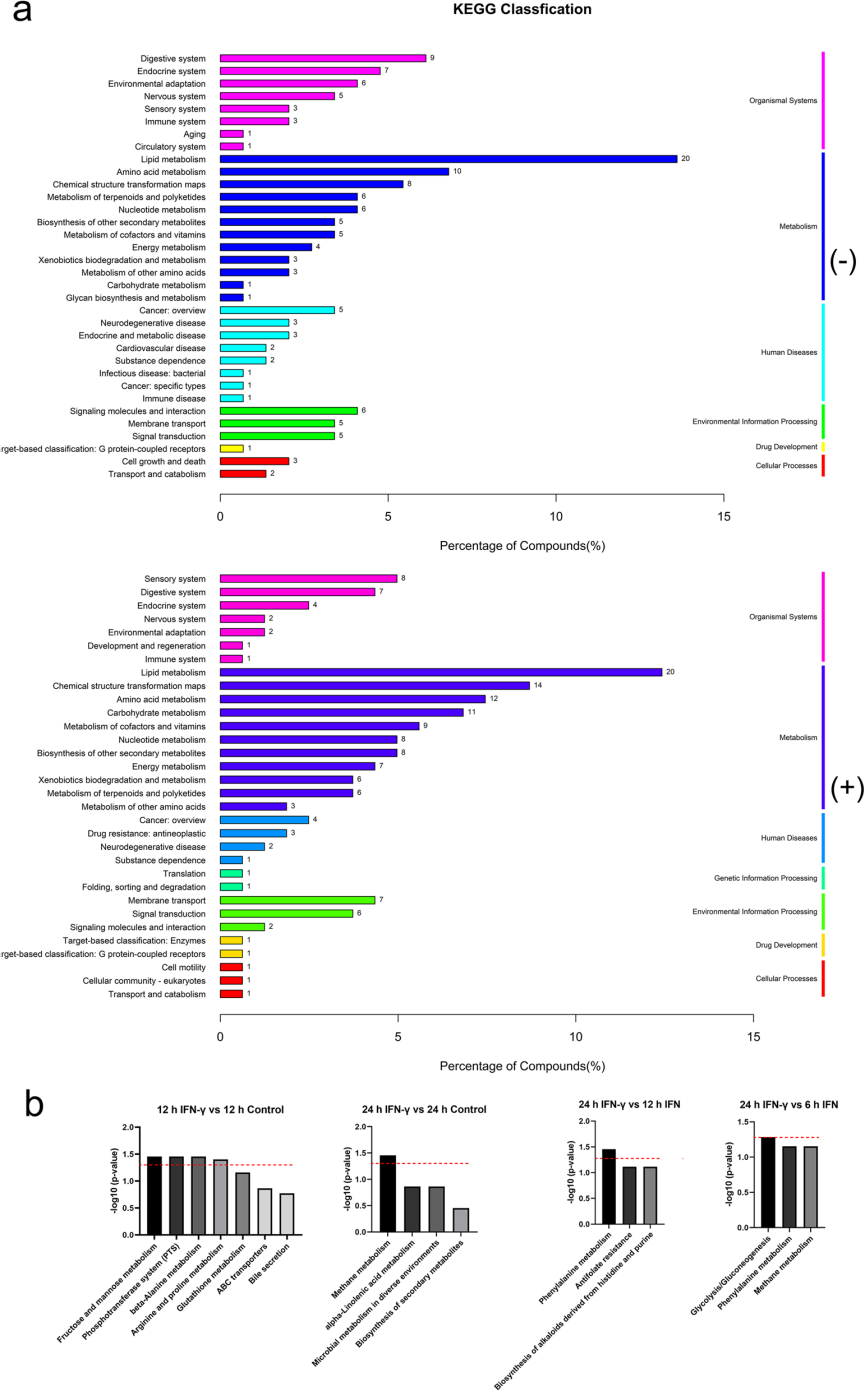
KEGG pathway enrichment analysis [21–23] based on differentially expressed metabolites (DEMs) upon IFN-γ treatment. (**a**) The KEGG classifications of the DEMs. The Y-axis indicates the KEGG terms. The number and degree of enrichment of pathways in a category is displayed in the X-axis. (**b**) The main enriched KEGG pathways of the DEMs. Red dotted lines indicate *p*-value = 0.05, which is defined as significant. (-), negative ion mode. ( +), positive ion mode

Subsequently, we analyzed the different metabolic pathways enrichment using the KEGG database [21–23]. The results showed that the DEMs were enriched in 141 and 115 KEGG metabolic pathways in negative and positive ion modes, respectively. The different metabolic classifications upon IFN-γ treatment are mainly enriched in category metabolism including lipid metabolism, amino acid metabolism, chemical structure transformation maps, nucleotide metabolism, and metabolism of cofactors and vitamins; organismal systems including digestive system; human diseases including cancer; environmental information processing including membrane transport (Fig. 5a). We then analyzed the top pathways with significant KEGG enrichment in both ion modes. Interestingly, many of the DEMs are not annotated in KEGG database. Specifically, the pathways of DEMs in IFN-γ treatment for 12 h group were mainly concentrated in fructose and mannose metabolism (00051, Fig. S3), phosphotransferase system (PTS) (02060, Fig. S4), β-alanine metabolism (00410, Fig. S5), arginine and proline metabolism (00330, Fig. S6), compared to control group; while that in IFN-γ treatment for 24 h group were only methane metabolism (00680, Fig. S7) (Fig. 5b). The pathways of the DEMs in IFN-γ treatment 24 h and 12 h groups were mainly concentrated in phenylalanine metabolism (00360, Fig. S8), while those of the DEMs in IFN-γ treatment 24 h and 6 h groups were mainly concentrated in glycolysis/gluconeogenesis (00010, Fig. S9) (Fig. 5b). Overall, these results demonstrated that IFN-γ treatment led to significant metabolic changes, especially lipid and amino acid metabolism in BMECs.

### Analysis of the DEMs in enriched pathways

**Fig. 6:**
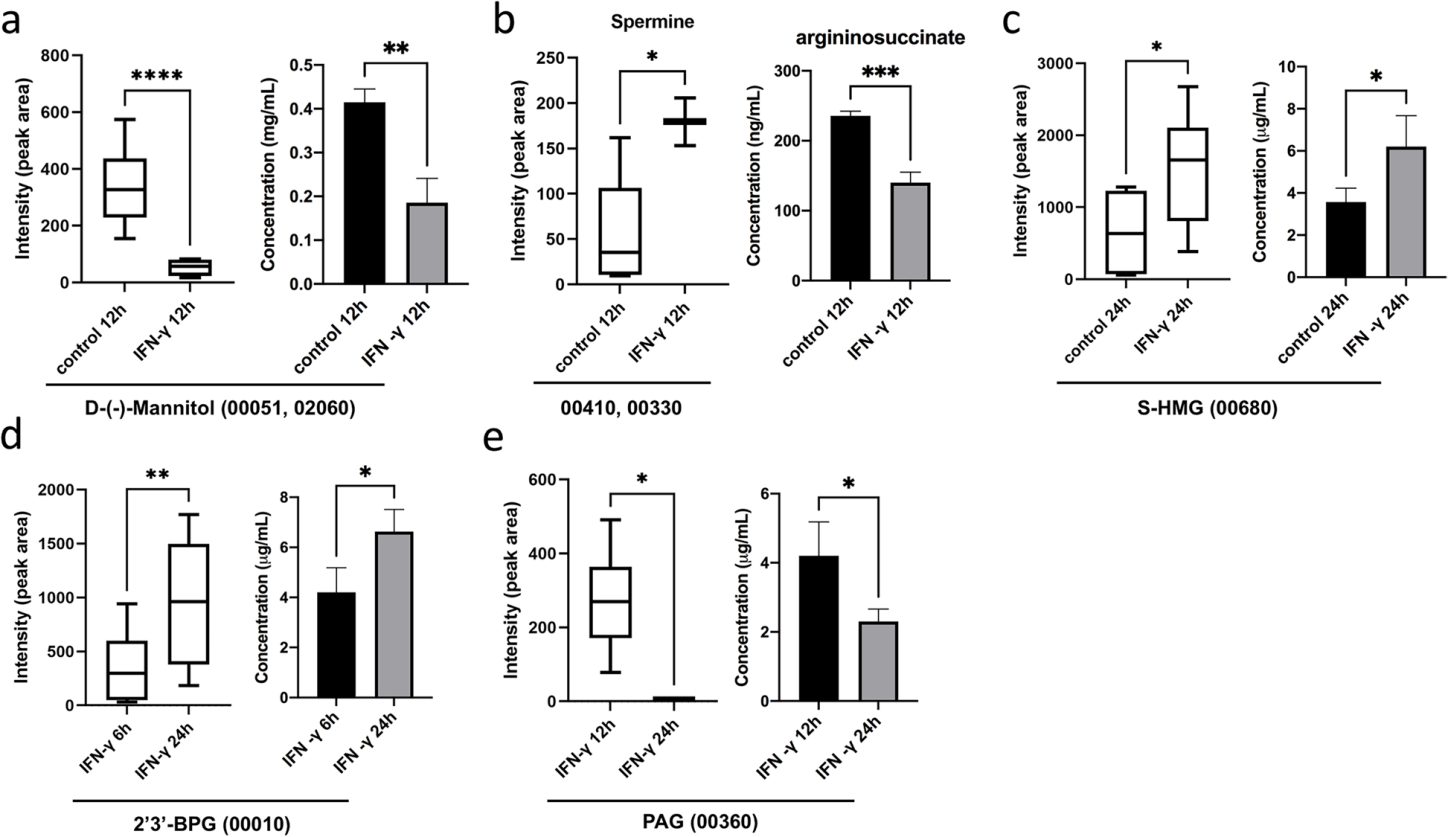
Quantification of the DEMs enriched in seven important pathways by targeted metabolomics. 00051, mannose metabolism pathway; 02060, phosphotransferase system (PTS) pathway; 00410, β-alanine metabolism pathway; 00330, arginine and proline metabolism pathway; 00680, methane metabolism pathway; 00010, glycolysis/gluconeogenesis pathway; 00360, phenylalanine metabolism pathway. Differences between mean values were assessed by two-tailed Student’s *t*-test. *p < 0.05; **p < 0.01; ***p < 0.001

To further analyze the DEMs enriched by KEGG classification, the contents of DEMs in significant altered pathways of each group were evaluated by enriched analysis and targeted metabolomics. All the significantly altered pathways comprised only one metabolite each (Fig. 6). Specifically, the content of D-(-)-Mannitol, in both the fructose and mannose metabolism (00051, Fig. S3) and phosphotransferase system (PTS) (02060, Fig. S4) pathways, was significantly decreased upon IFN-γ treatment for 24 h compared with control in MAC-T cells (Fig. 6A).

On the other hand, the content of spermine, in both the β-alanine metabolism (00410, Fig. S5) and arginine and proline metabolism (00330, Fig. S6) pathways, was significantly increased upon IFN-γ treatment for 12 h compared with control in MAC-T cells (Fig. 6A, Fig. S7). Previously we have shown that IFN-γ treatment did not affect ornithine level, but led to reduced intracellular levels of arginine and citrulline (Fig. S7), two of which are the key metabolites for arginine metabolism [25]. As a vital intermediate product for arginine synthesis, we further detected the intracellular level of argininosuccinate in this study. We found that the intracellular level of argininosuccinate was significantly reduced upon IFN-γ treatment (Fig. 6B), suggesting IFN-γ interferes with arginine synthesis by downregulation of argininosuccinate production in MAC-T cells.

Similarly, the content of S-hydroxymethylglutathione (S-HMG) and 2,3-bisphospho-D-glyceric acid (2,3-BPG), in methane metabolism (00680, Fig. S8) and glycolysis/gluconeogenesis (00010, Fig. S10) pathway, respectively, was also significantly increased upon IFN-γ treatment for 24 h (Fig. 6C and D). Lastly, the content of phenylacetylglycine (PAG) in phenylalanine metabolism (00360, Fig. S9) pathway was significantly decreased upon IFN-γ treatment for 24 h compared with IFN-γ treatment for 12 h (Fig. 6E).

## Discussion

In this study, untargeted and targeted metabolomics were performed using UPLC-TOF/MS to explore the mechanism for IFN-γ induced arginine depletion in BMECs. Results showed that IFN-γ induction resulted in significant differences in the contents of metabolites. KEGG pathway analysis demonstrated that most of the altered pathways were those associated with fructose and mannose metabolism, phosphotransferase system (PTS), β-alanine metabolism, arginine and proline metabolism, methane metabolism, phenylalanine metabolism, and glycolysis/gluconeogenesis.

Fructose and mannose metabolism are one of the most altered pathways in BMECs upon IFN-γ induction. We found that the content of D-(-)-Mannitol, a metabolite involves in both fructose and mannose metabolism and PTS, was significantly decreased (Fig. 6A; Fig. S3). Mannitol is widespread in both eukaryotic and prokaryotic life as a sugar or sugar alcohol. In clinical settings, mannitol has been utilized as a highly effective dehydrating agent and osmotic diuretic that contributes to minimize the risk of acute renal failure in patients after renal transplantation [26]. It facilitates excretion of water and toxic materials of tubular epithelial cells. Mannitol is also indicated as add-on maintenance therapy for improving pulmonary function in cystic fibrosis patients [27]. It is hypothesized that mannitol produces an osmotic gradient across the airway epithelium that draws fluid into the extracellular space and alters the properties of the airway surface mucus layer, which allows easier mucociliary clearance [27]. Previous results from our group demonstrated that IFN-γ induced malignant transformation of BMECs [16, 28], a precancerous phenotype with drastic cell morphology and function alternations. We assume that the downregulation of mannitol induced by IFN-γ might alters osmosis of BMECs, which leads to accumulation of toxic materials that interfere with cell normal functionality.

Spermine is one of the metabolites involve in both the β-alanine metabolism and arginine and proline metabolism pathways (Fig. 6B; Fig. S5, S6). Spermine belongs to polyamines and it is synthesized from arginine and s-adenosylmethionine [29]. It is involved in diverse functions including cell growth and differentiation in terms of DNA synthesis and stability, regulation of transcription, ion channel regulation, and protein phosphorylation [29, 30]. Notably, spermine enhances cell growth and thus the biosynthesis spermine is upregulated in cancer cells [31]. In BMECs, IFN-γ accelerates cell growth and induces malignant transformation through arginine depletion [16, 28]. It’s been shown that IFN-γ could disturb arginine metabolism by affecting the expression of key time-limiting enzymes ASS1 [25, 32]. In this study, the content of spermine was downregulated, which further confirms these results and suggest that the IFN-γ-induced malignant transformation of BMECs might possibly be associated with increased spermine levels in cells.

S-hydroxymethylglutathione (S-HMG) is the spontaneous adduct of formaldehyde and glutathione. It is oxidized by S-nitrosoglutathione reductase (GSNOR) to S-formylglutathione (FGSH) in cells [33]. It’s been shown that GSNOR plays an important regulatory role in smooth muscle relaxation, immune function, inflammation, neuronal development, and cancer progression [33]. In addition, GSNOR also modulates the availability of intracellular reactive nitric oxide, a molecule which functions in inflammation and cancer immunity [34]. Of note, S-HMG plays a vital role in the detoxication of formaldehyde [35]. In this study, the level of S-HMG was significantly increased upon IFN-γ induction (Fig. 6C), indicating a possible accumulation of formaldehyde in BMECs upon IFN-γ induction that might be associated with the malignant transformation of BMECs. However, whether S-HMG affects arginine metabolism or vice versa stills need to be further demonstrated.

2,3-bisphospho-D-glyceric acid (ENO1) is one of the metabolites in glycolysis/gluconeogenesis pathway (Fig. S10). It is a glycolytic enzyme that catalyzes the conversion of 2-phosphoglyceric acid to phosphoenolpyruvic acid during glycolysis [36]. Glycolysis/gluconeogenesis plays vital roles in tumorigenesis where ENO1 involves in. It has been shown that ENO1 expression was enhanced in many tumor cells [37]. ENO1 contributes to tumorigenesis by promotion of tumor proliferation, inhibition of cancer cell apoptosis, invasion and metastasis of tumor cells [36]. Interestingly, the content of ENO1 was also upregulated upon IFN-γ induction in BMECs (Fig. 6D), indicating that ENO1 might also involves in IFN-γ-induced malignant transformation of BMECs.

Lastly, the content of phenylacetylglycine in phenylalanine metabolism was significantly downregulated upon IFN-γ induction in BMECs (Fig. 6E; Fig. S9). Phenylacetylglycine is a terminal product of phenylalanine metabolism and accepted as a biomarker for phospholipidosis [38], diabetes [39], and prostate cancer [40]. However, there is limited information about the correlation between phenylacetylglycine and arginine metabolism or tumorigenesis. Thus, phenylacetylglycine might involves in IFN-γ induced arginine depletion and malignant transformation of BMECs indirectly.

In conclusion, our study reveals potential metabolites and signaling pathways in BMECs upon IFN-γ induction. IFN-γ induces arginine depletion and malignant transformation of BMECs possibly through modulation of arginine metabolism, cell osmosis, and metabolites associated with tumorigenesis, including S-HMG and ENO1. These results provide potential theoretical basis for clarifying mechanism of diseases due to abnormal IFN-γ level.

## Supplementary Information

**Additional file 1:** **Figure S1.** Validation of untargeted metabolomics using quality control (QC) samples. The correlation heat map showed the correlation coefficient among QC samples in both ionization modes.

**Additional file 2:** **Figure S2.** The permutation test of the orthogonal partial least squares discriminant analysis (OPLS-DA) model. The arrows show the results from the data compared with frequency histograms of the scores from 1000 permutations of the data which show the expected distribution of scores if no association exists. The x-axis represents the accuracy of the model. The y-axis represents the frequency of the model accuracy from 1000 permutations of the data.

**Additional file 3:** **Figure S3.** KEGG pathway for fructose and mannose metabolism. The differentially expressed metabolite (DEM), D-(-)-Mannitol, is highlighted in green.

**Additional file 4:** **Figure S4.** KEGG pathway for phosphotransferase system (PTS). The differentially expressed metabolite (DEM), D-(-)-Mannitol, is highlighted in green.

**Additional file 5:** **Figure S5.** KEGG pathway for β-alanine metabolism. The differentially expressed metabolite (DEM), spermine, is highlighted in red.

**Additional file 6:** **Figure S6.** KEGG pathway for arginine and proline metabolism. The differentially expressed metabolite (DEM), spermine, is highlighted in red.

**Additional file 7:** **Figure S7.** Quantification of key metabolites involved in arginine metabolism by targeted metabolomics. Differences between mean values were assessed by two-tailed Student’s *t*-test. *p < 0.05; **p < 0.01; ***p < 0.001.

**Additional file 8:** **Figure S8.** KEGG pathway for methane metabolism. The differentially expressed metabolite (DEM), S-hydroxymethylglutathione (S-HMG), is highlighted in red.

**Additional file 9:** **Figure S9.** KEGG pathway for phenylalanine metabolism. The differentially expressed metabolite (DEM), phenylacetylglycine, is highlighted in green.

**Additional file 10:** **Figure S10.** KEGG pathway for glycolysis/gluconeogenesis. The differentially expressed metabolite (DEM), 2,3-bisphospho-D-glyceric acid (2,3-BPG or glycerate-2,3P_2_), is highlighted in red

**Additional file 11:** **Table S1.** Summary of the number of differentially expressed metabolites (DEMs) in each comparison group in both negative and positive ion modes

**Additional file 12:** **Table S2.** List of differentially expressed metabolites (DEMs) upon 6 h IFN-γ treatment compared to control in both negative and positive ion modes.

**Additional file 13:** **Table S3.** List of differentially expressed metabolites (DEMs) upon 12 h IFN-γ treatment compared to control in both negative and positive ion modes.

**Additional file 14:** **Table S4.** List of differentially expressed metabolites (DEMs) upon 24 h IFN-γ treatment compared to control in both negative and positive ion modes.

**Additional file 15:** **Table S5.** List of differentially expressed metabolites (DEMs) upon 12 h IFN-γ treatment compared to 6 h IFN-γ treatment in both negative and positive ion modes.

**Additional file 16:** **Table S6.** List of differentially expressed metabolites (DEMs) upon 24 h IFN-γ treatment compared to 6 h IFN-γ treatment in both negative and positive ion modes.
**Additional file 17:** **Table S7.**  List of differentially expressed metabolites (DEMs) upon 24 h IFN-γ treatment compared to 12 h IFN-γ treatment  in both negative and positive ion modes.

## Data Availability

Different metabolites expression profiles identified by untargeted metabolomics in each comparison group has been deposited in Zenodo (https://doi.org/10.5281/zenodo.5880761; https://doi.org/10.5281/zenodo.5880787) and summarized information has been uploaded to Figshare (https://doi.org/10.6084/m9.figshare.20898301.v1). All other data supporting the conclusions of this article is included within the article and its additional file.

## Abbreviations

BMECs: Bovine mammary epithelial cells
UPLC-MS: Ultra-performance liquid chromatography-mass spectrometry
DEMs: Differentially expressed metabolites
KEGG: Kyoto Encyclopedia of Genes and Genomes
PTS: Phosphotransferase system
NO: Nitric oxide
QC: Quality control
PCA: Principal component analysis
OPLS-DA: Orthogonal partial least squares discriminant analysis
HMDB: Human Metabolome Database
LMSD: LIPID MAPS Structure Database
S-HMG: S-hydroxymethylglutathione
2,3-BPG: 2,3-Bisphospho-D-glyceric acid
