# Supplementary material for: Untargeted metabolomics reveals alternations in metabolism of bovine mammary epithelial cells upon IFN-γ treatment
Source: BMC Vet Res. 2023 Feb 11;19:44. doi: 10.1186/s12917-023-03588-2 (PMC9921584; doi:10.1186/s12917-023-03588-2)
Supplement: Supplementary file 1 — Additional file 1: Figure S1. Validation of untargeted metabolomics using quality control (QC) samples. The correlation heat map showed the correlation coefficient among QC samples in both ionization modes. [file 12917_2023_3588_MOESM1_ESM.docx]

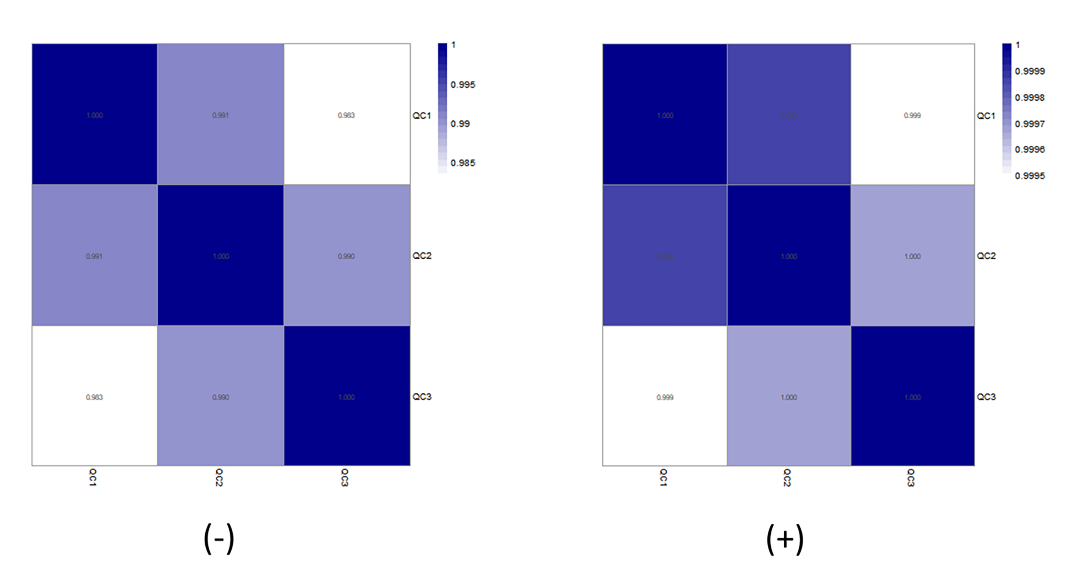


**Figure S1.** Validation of untargeted metabolomics using quality control (QC) samples. The correlation heat map showed the correlation coefficient among QC samples in both ionization modes.
