# Supplementary material for: Untargeted metabolomics reveals alternations in metabolism of bovine mammary epithelial cells upon IFN-γ treatment
Source: BMC Vet Res. 2023 Feb 11;19:44. doi: 10.1186/s12917-023-03588-2 (PMC9921584; doi:10.1186/s12917-023-03588-2)
Supplement: Supplementary file 2 — Additional file 2: Figure S2. The permutation test of the orthogonal partial least squares discriminant analysis (OPLS-DA) model. The arrows show the results from the data compared with frequency histograms of the scores from 1000 permutations of the data which show the expected distribution of scores if no association exists. The x-axis represents the accuracy of the model. The y-axis represents the frequency of the model accuracy from 1000 permutations of the data. [file 12917_2023_3588_MOESM2_ESM.docx]

**
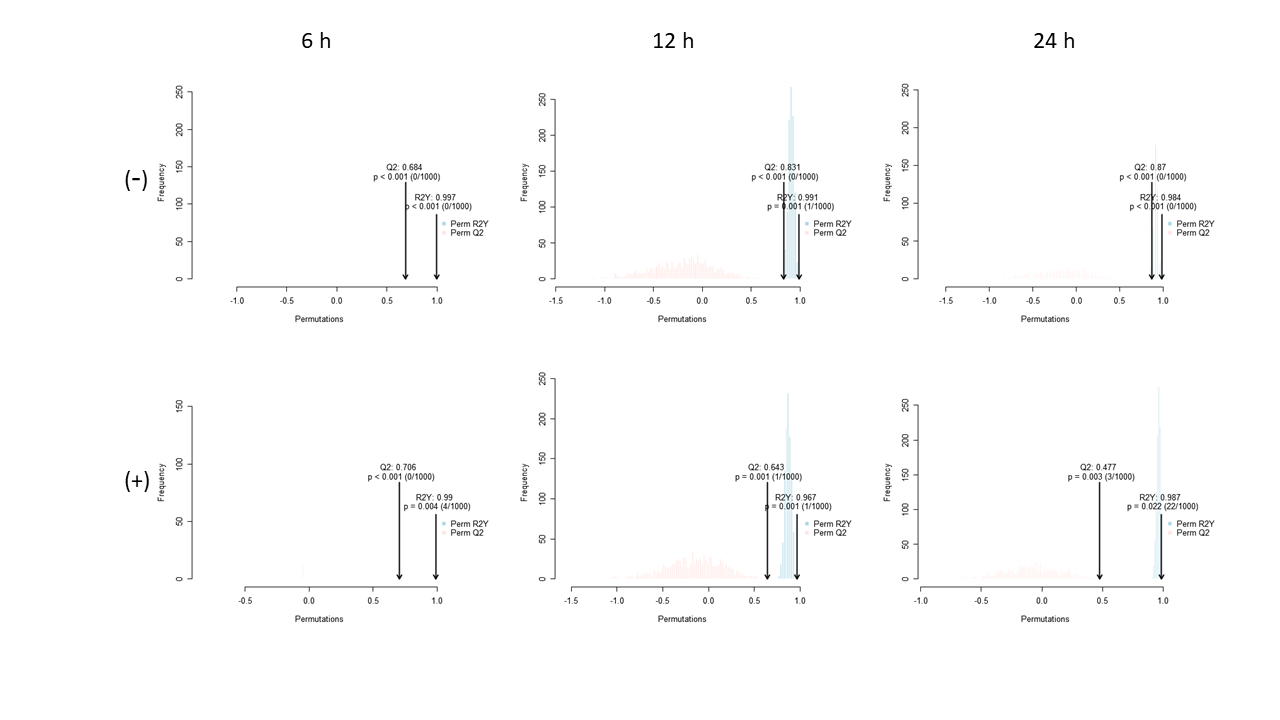
**

**Figure S2.** The permutation test of the orthogonal partial least squares discriminant analysis (OPLS-DA) model. The arrows show the results from the data compared with frequency histograms of the scores from 1000 permutations of the data which show the expected distribution of scores if no association exists. The x-axis represents the accuracy of the model. The y-axis represents the frequency of the model accuracy from 1000 permutations of the data.
