# Supplementary material for: Untargeted metabolomics reveals alternations in metabolism of bovine mammary epithelial cells upon IFN-γ treatment
Source: BMC Vet Res. 2023 Feb 11;19:44. doi: 10.1186/s12917-023-03588-2 (PMC9921584; doi:10.1186/s12917-023-03588-2)
Supplement: Supplementary file 4 — Additional file 4: Figure S4. KEGG pathway for phosphotransferase system (PTS). The differentially expressed metabolite (DEM), D-(-)-Mannitol, is highlighted in green. [file 12917_2023_3588_MOESM4_ESM.docx]

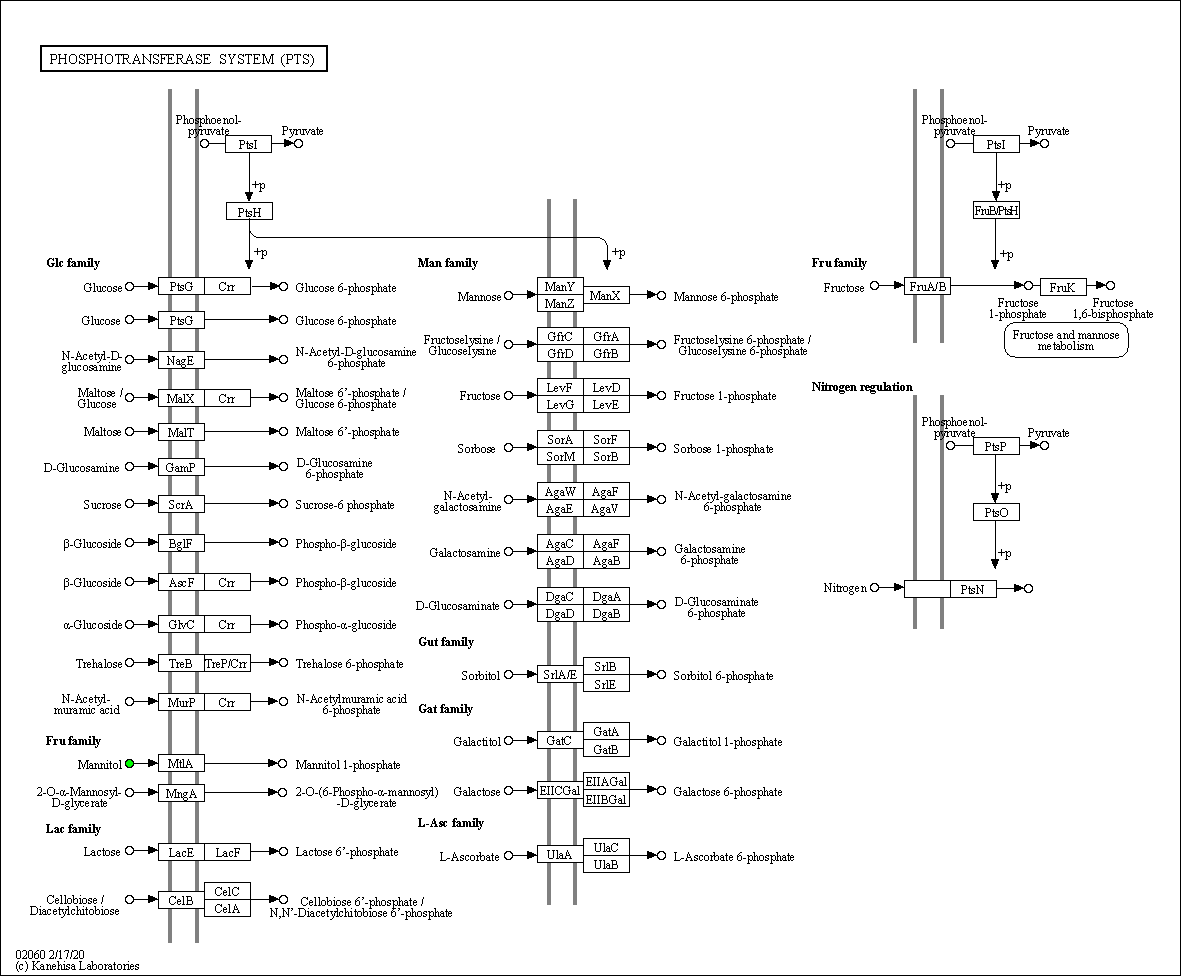


**Figure S4.** KEGG pathway for phosphotransferase system (PTS). The differentially expressed metabolite (DEM), D-(-)-Mannitol, is highlighted in green.
