# Supplementary material for: Untargeted metabolomics reveals alternations in metabolism of bovine mammary epithelial cells upon IFN-γ treatment
Source: BMC Vet Res. 2023 Feb 11;19:44. doi: 10.1186/s12917-023-03588-2 (PMC9921584; doi:10.1186/s12917-023-03588-2)
Supplement: Supplementary file 5 — Additional file 5: Figure S5. KEGG pathway for β-alanine metabolism. The differentially expressed metabolite (DEM), spermine, is highlighted in red. [file 12917_2023_3588_MOESM5_ESM.docx]

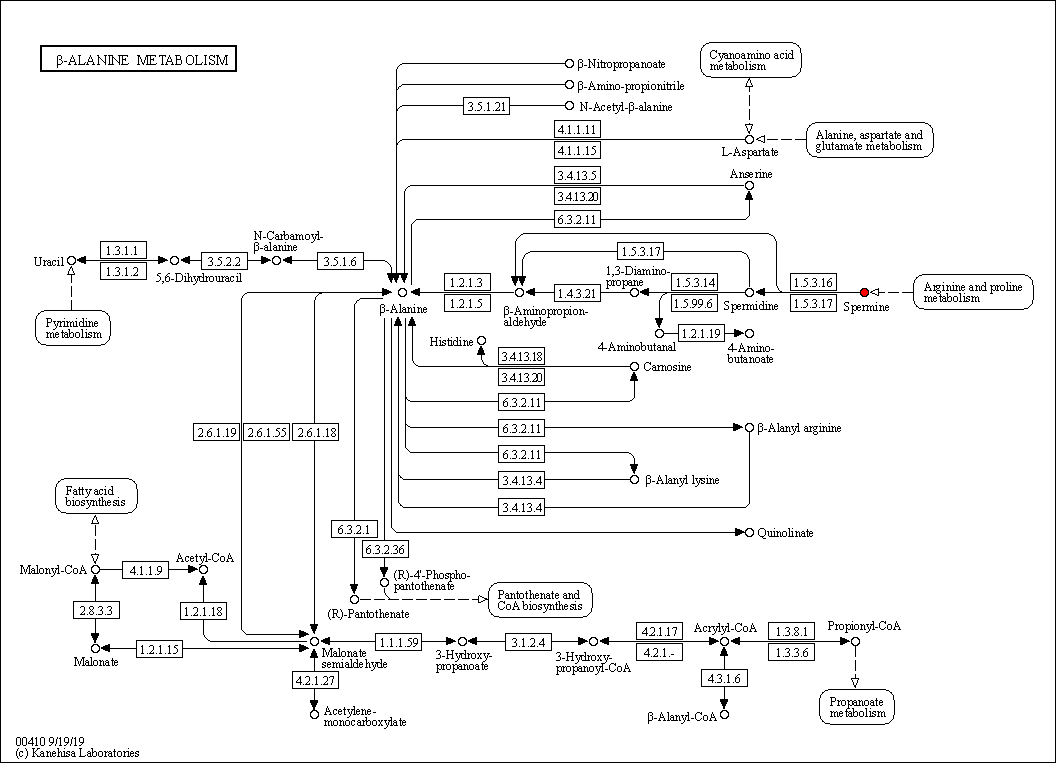


**Figure S5.** KEGG pathway for β-alanine metabolism. The differentially expressed metabolite (DEM), spermine, is highlighted in red.
