# Supplementary material for: Untargeted metabolomics reveals alternations in metabolism of bovine mammary epithelial cells upon IFN-γ treatment
Source: BMC Vet Res. 2023 Feb 11;19:44. doi: 10.1186/s12917-023-03588-2 (PMC9921584; doi:10.1186/s12917-023-03588-2)
Supplement: Supplementary file 7 — Additional file 7: Figure S7. Quantification of key metabolites involved in arginine metabolism by targeted metabolomics. Differences between mean values were assessed by two-tailed Student’s t-test. *p < 0.05; **p < 0.01; ***p < 0.001. [file 12917_2023_3588_MOESM7_ESM.docx]

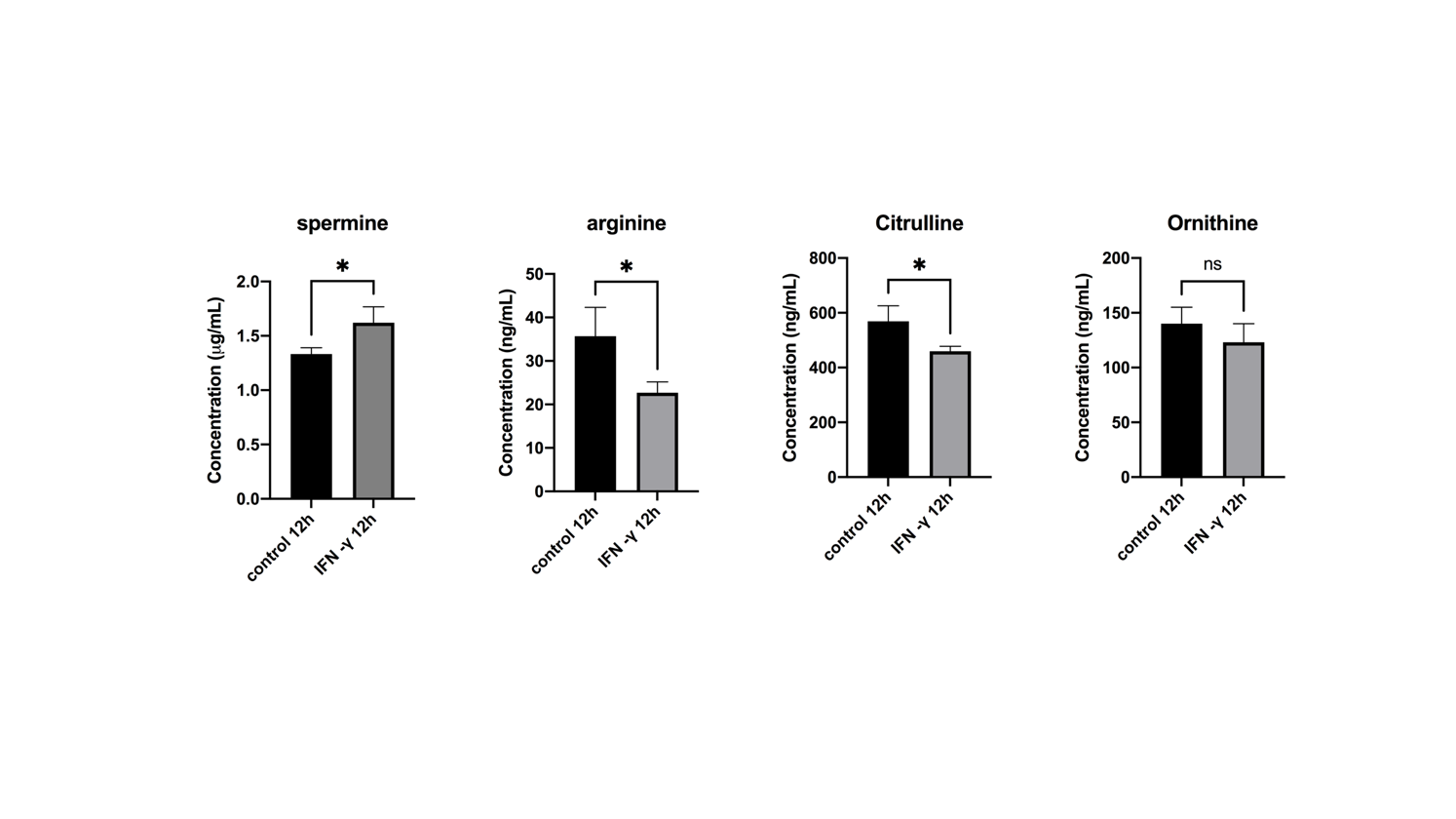


**Figure S7.** Quantification of key metabolites involved in arginine metabolism by targeted metabolomics. Differences between mean values were assessed by two-tailed Student’s *t*-test. **p* < 0.05; ***p* < 0.01; ****p* < 0.001.
