# Supplementary material for: Untargeted metabolomics reveals alternations in metabolism of bovine mammary epithelial cells upon IFN-γ treatment
Source: BMC Vet Res. 2023 Feb 11;19:44. doi: 10.1186/s12917-023-03588-2 (PMC9921584; doi:10.1186/s12917-023-03588-2)
Supplement: Supplementary file 10 — Additional file 10: Figure S10. KEGG pathway for glycolysis/gluconeogenesis. The differentially expressed metabolite (DEM), 2,3-bisphospho-D-glyceric acid (2,3-BPG or glycerate-2,3P2), is highlighted in red [file 12917_2023_3588_MOESM10_ESM.docx]

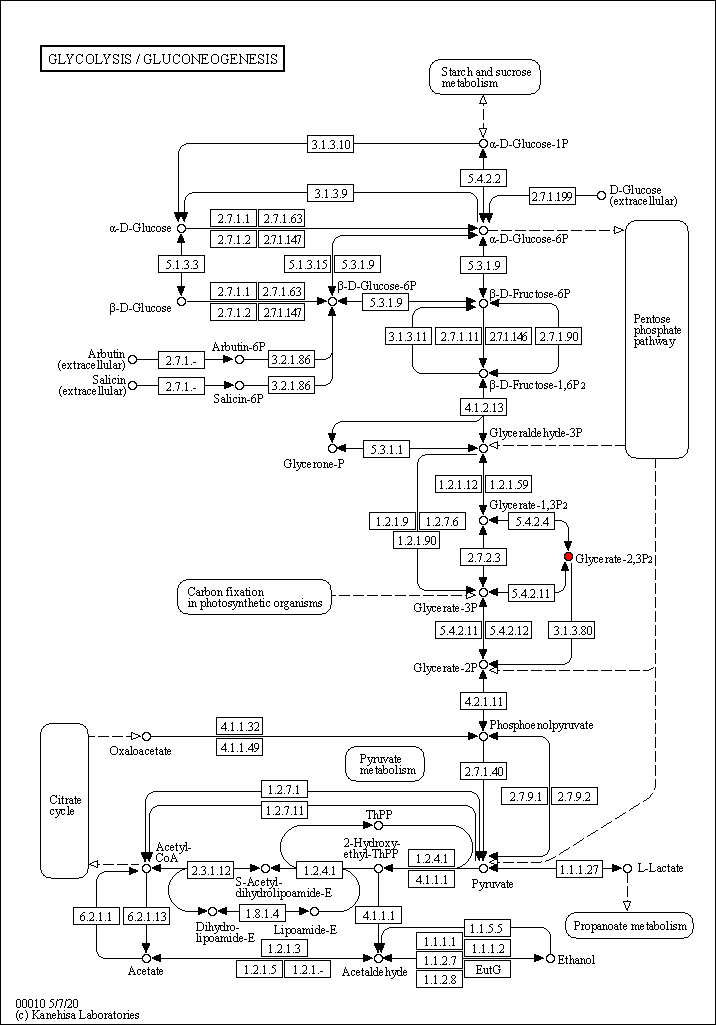


**Figure S9.** KEGG pathway for glycolysis/gluconeogenesis. The differentially expressed metabolite (DEM), 2,3-bisphospho-D-glyceric acid (2,3-BPG or glycerate-2,3P_2_), is highlighted in red.
